# A systematic review of generational preferences of telepsychiatry versus in person psychiatry visits

**DOI:** 10.1192/j.eurpsy.2026.12062

**Published:** 2026-07-31

**Authors:** R. Jankay, W. Amor, R. Baronia, C. Studzinki, S. Suduth

**Affiliations:** Texas Tech University Health Science Center, Lubbock, United States

## Abstract

**Introduction:**

Psychiatric care delivery has evolved given the growth of telepsychiatry during the pandemic. Thus, it is vital to understand patient and generational preferences in order to best guide care and optimize resources. This review explores valuable literature on patient attitudes toward telepsychiatry and in-person psychiatry across different age groups, identifying satisfaction levels, outcomes, and other influencing factors.

**Objectives:**

1. Identify research on patient preferences for telepsychiatry versus in-person psychiatry.
2. Compare overall outcomes based on age and patient demographic characteristics.
3. Review reported satisfaction levels in relation to visit modality.

**Methods:**

A systematic search was conducted using PubMed, Embase, CINAHL, and PsycINFO databases. COVIDENCE software was used for data management. Inclusion criteria encompassed peer-reviewed studies reporting patient preferences or satisfaction with telepsychiatry and/or in-person psychiatry in outpatient settings. Some search terms examples included “Young adults (19-24), Generation Z... to Aged (65-79), Generation Y.” Exclusion criteria included inpatient psychiatric settings and studies lacking generational or age-group analysis. Initially, 126 articles were screened, after filtering 46 full-text articles were reviewed. Extraction data included criteria such as study year, population demographics, visit modality, and reported outcomes.

**Results:**

Initial analysis indicates the younger generation, mostly millennials, have higher preference for telepsychiatry modalities, due to convenience, less travel time, and added flexibility. Middle-aged and older adults had mixed preferences, rapport building and higher quality communication was noted during in-person visits. Overall, satisfaction was high for both modalities after certain privacy concerns were adjusted for. The majority of studies showed no significant difference in clinical outcomes between modalities, though modality choice impacted patient engagement

**Conclusions:**

Differences exist and impact patient preferences in psychiatry care delivery modes. Those in younger cohorts tend to prefer telepsychiatry and those in older cohorts have more variety in their preferences. Both show high satisfaction and similar outcomes when delivered properly. These findings highlight the significance of providing flexible options that take into account age and technology comfort levels of patients. Further research will enable deeper understanding in telepsychiatry accessibility and acceptability among various unique populations.

**Disclosure of Interest:**

None Declared

